# Smad4 Deficiency in S100A4^+^ Macrophages Enhances Colitis-associated Tumorigenesis by Promoting Macrophage Lipid Metabolism Augmented M2 Polarization

**DOI:** 10.7150/ijbs.98529

**Published:** 2024-11-11

**Authors:** Ting Liu, Xinyuan Zhang, Xuanxuan Yan, Leirong Cheng, Xinlong Yan, Fanxin Zeng, Xue Li, Zhinan Chen, Jianchun Gu, Jinhua Zhang

**Affiliations:** 1 School of Life Science and Technology, Jinan University, Guangzhou, Guangdong Province, P.R. China.; 2 The College of Life Science and Bioengineering, Beijing Jiaotong University, Beijing, P.R. China.; 3 State Key Laboratory of Targeting Oncology, Guangxi Medical University, Nanning, Guangxi Province, P.R. China.; 4 Faculty of Environmental and Life Sciences, Beijing University of Technology, Beijing, P.R. China.; 5 Department of Clinical Research Center, Dazhou Central Hospital, Dazhou, Sichuan Province, P.R. China.; 6 National Translational Science Center for Molecular Medicine & Department of Cell Biology, Fourth Military Medical University, Xian, Shanxi Province, P.R. China.; 7 Department of Oncology, Xinhua Hospital Affiliated to Shanghai Jiaotong University School of Medicine, Shanghai, P.R. China.

**Keywords:** S100A4, Smad4, Fabp2, STAT6, macrophage, metabolism.

## Abstract

S100A4 is primarily expressed in intestinal macrophages, and promotes colonic inflammation and colitis-associated colon tumorigenesis. Smad4 is also expressed in the colon; however, it inhibits colitis-associated cancer (CAC) development. The specific role of Smad4 in S100A4^+^ cells in CAC remains unknown. In this study, an azoxymethane (AOM)/dextran sodium sulfate (DSS)-induced CAC model was established in mice with S100A4^+^ cell-specific Smad4 deletion (S100A4^ Smad4-/-^). Smad4 deficiency in S100A4^+^ cells exacerbated DSS-induced colitis and promoted colorectal tumorigenesis. In addition, S100A4^+^ cell-specific Smad4 ablation promoted the M2 polarization of macrophages in CAC. Mechanistically, Smad4 depletion in macrophages enhanced lipid metabolism by activating the FA binding protein 2 (Fabp2)/STAT6 pathway. Furthermore, Smad4 deficiency in macrophages promoted MC38 tumor growth in myeloid-specific Smad4 deficient (Lyz^ Smad4-/-^) mice, whereas blocking Fabp2 expression reversed the tumor growth. Additionally, high Smad4 expression was associated with prolonged survival in patients with colorectal cancer. Thus, Smad4 in S100A4^+^ macrophages plays a tumor-inhibiting role in CAC development and supports its use as a prognostic marker in CRC patients.

## Background

Chronic inflammation is essential for the development of various tumors, including gastric carcinoma, lung cancer, and colorectal cancer (CRC) 1, 2. CRC is the second leading cause of cancer death worldwide and is a major health concern because of its high incidence and mortality 3. Patients with inflammatory bowel diseases (IBD), including ulcerative colitis (UC) and Crohn's disease (CD), are more likely to develop CRC 4, 5. A recent meta-analysis revealed the overall prevalence risks for CRC were approximately 0.02% at 10 years, 4.81% at 20 years, and 13.91% at 30 years 6. However, the detailed molecular mechanism underlying inflammation-associated colorectal tumorigenesis has not yet been fully explored.

The development of CRC is accompanied with the infiltration of several immune cells, including macrophages, T cells, NK cells, and neutrophils 7, which are continuously recruited to the tumor mass to form a complex tumor microenvironment (TME). Tumor-associated macrophages (TAMs), one of the most abundant immune cells in the TME promote the initiation and metastasis of tumor cells, inhibit the T cell-mediated anti-tumor immune responses, and induce tumor angiogenesis and subsequently tumor progression. TAMs are highly plastic and heterogeneous. M2-polarized TAMs are important regulators of the link between inflammation and cancer and are critical modulators of the TME 8.

S100 calcium-binding protein A4 (S100A4), also known as FSP1, or metastasin, is a member of the S100 family. S100A4 is expressed in a variety of cells, such as fibroblasts, macrophages, and tumor cells 9, 10. It promotes tumorigenesis and metastasis through increased cell proliferation, invasion, and angiogenesis 11, 12. S100A4 expression is increased in human CRC tissues 13 and 82% of patients with CRC exhibit increased S100A4 mRNA expression levels 14. Furthermore, S100A4 was shown to be a prognostic marker for CRC 15. S100A4 overexpression in CRC tissues is associated with poor survival of patients 16. Consistently, our study using S100A4-deficient mice revealed that S100A4 exacerbated an inflammatory microenvironment to promote colon tumorigenesis 17, 18.

Smad4 belongs to the SMAD family and is a key signal transduction molecule in the TGF-β signaling pathway 19. It regulates the cell cycle, metabolism, transport, and other processes 20. As a tumor suppressor, Smad4 plays an important role in colorectal carcinogenesis and invasiveness 21. Smad4 loss in CRC organoids induces C-MYC-mediated NLE1 upregulation to support CRC growth and metastasis 22. Deficiency of Smad4 in the colon epithelium increases CCL20 expression and chemoattraction of CCR6^+^ immune cells, thus promoting colitis-associated carcinogenesis 23. Little is known regarding the effects of Smad4 in S100A4^+^ cells during colitis-associated cancer (CAC). In the present study, we explored the specific role of Smad4 in S100A4^+^ cells during colitis-associated carcinogenesis using S100A4^+^ cell-specific Smad4 deletion (S100A4^Smad4-/-^) mice and found that Smad4-specific deletion in S100A4^+^ cells promoted colitis-associated colon carcinogenesis, suggesting a tumor-inhibiting role of Smad4 signaling in S100A4^+^ cells.

## Results

### Smad4 expression in S100A4^+^ macrophages is down-regulated in colitis and colitis-associated CRC

**Figure 1:**
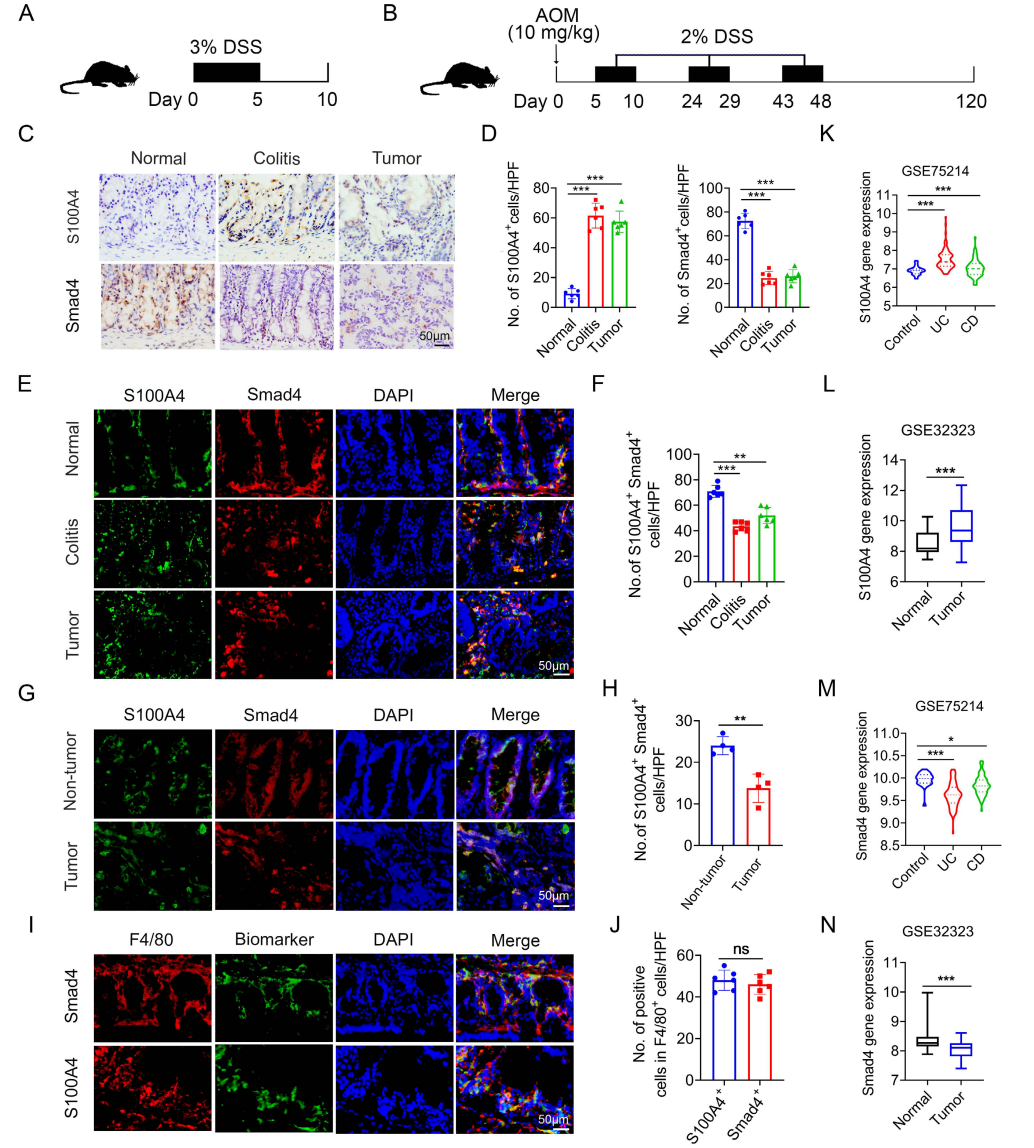
** Smad4 expression in S100A4^+^ macrophages is down-regulated in colitis and colitis-associated colorectal cancer.** C57BL/6 mice (n = 6 per group) were used for establishing dextran sodium sulfate (DSS)-induced colitis and azoxymethane (AOM) /DSS-induced colitis-associated cancer (CAC). The data are representative of three independent experiments. **(A-B)** Schematic representation of DSS-induced colitis and AOM/DSS-induced CAC model. **(C)** Representative staining of S100A4 and Smad4 in colon specimens (scale bars: 50 μm) and **(D)** statistical analysis. **P < 0.01. **(E)** Double immunohistochemical staining (IF) of S100A4 and Smad4 in normal and AOM/DSS-induced colon tissues (Scale bar: 50 μm) and **(F)** statistical analysis. **P < 0.01, ***P < 0.001. **(G)** Representative double staining for S100A4 and Smad4 in human colorectal cancer (CRC) tissues (Scale bar: 50 μm) and **(H)** statistical analysis. **P < 0.01. **(I)** Representative double staining of F4/80 and Smad4 in DSS-induced colon specimens (scale bars: 50 μm) and **(J)** statistical analysis. Violin diagram and Boxplots showing the expression levels of **(K-L)** S100A4 and **(M-N)** Smad4 in the colon dataset GSE75214 and GSE32323. *P < 0.05, ***P < 0.001. No: number; HPF: high-power field.

To determine the role of Smad4 in S100A4^+^ cells in colorectal carcinogenesis, mouse models of DSS and AOM/DSS-induced acute colitis and colitis-associated cancer were established (Figures 1A, B). A histological assessment revealed a significant upregulation of S100A4 expression and down-regulation of Smad4 in both colitis and CRC tissues (Figures 1C, D). Smad4 expression in S100A4^+^ cells was also decreased in colitis and tumor tissues (Figures 1E, F). Similarly, the number of Smad4^+^/S100A4^+^ cells was significantly lower in tumors than in adjacent tissues of patients with CRC, as determined via double staining (Figures 1G, H). Consistently, GEO dataset analysis revealed that S100A4 expression was significantly increased in UC, CD, and colon cancer tissues compared with that in normal tissues (Figures 1K, L). Smad4 expression was down-regulated in UC, CD, and colon cancer tissues (Figures 1M, N). These results suggest that Smad4 in S100A4^+^ cells plays an active role in the prevention of colitis and colon cancer.

Previous studies have shown that S100A4 is expressed by diverse cells within the TME, including fibroblasts, macrophages, and T cells; however, S100A4^+^ cells constitute an inflammatory subpopulation of macrophages in the liver 24. Recent studies have demonstrated that most S100A4^+^ cells are macrophages in transplanted tumors 25, 26. In a previous study, we also found that most of the S100A4^+^ cells in colon tissues were not fibroblasts, but primarily myeloid cells, which express CD11b and F4/80 17. Using double staining, we revealed that most of the Smad4^+^ cells were F4/80^+^ macrophages in colon tissues (Figures 1I, J). However, it remains unclear whether Smad4 in S100A4^+^ cells affects colitis-associated carcinogenesis.

### Smad4 deficiency in S100A4^+^ cells aggravates DSS-induced colitis

**Figure 2:**
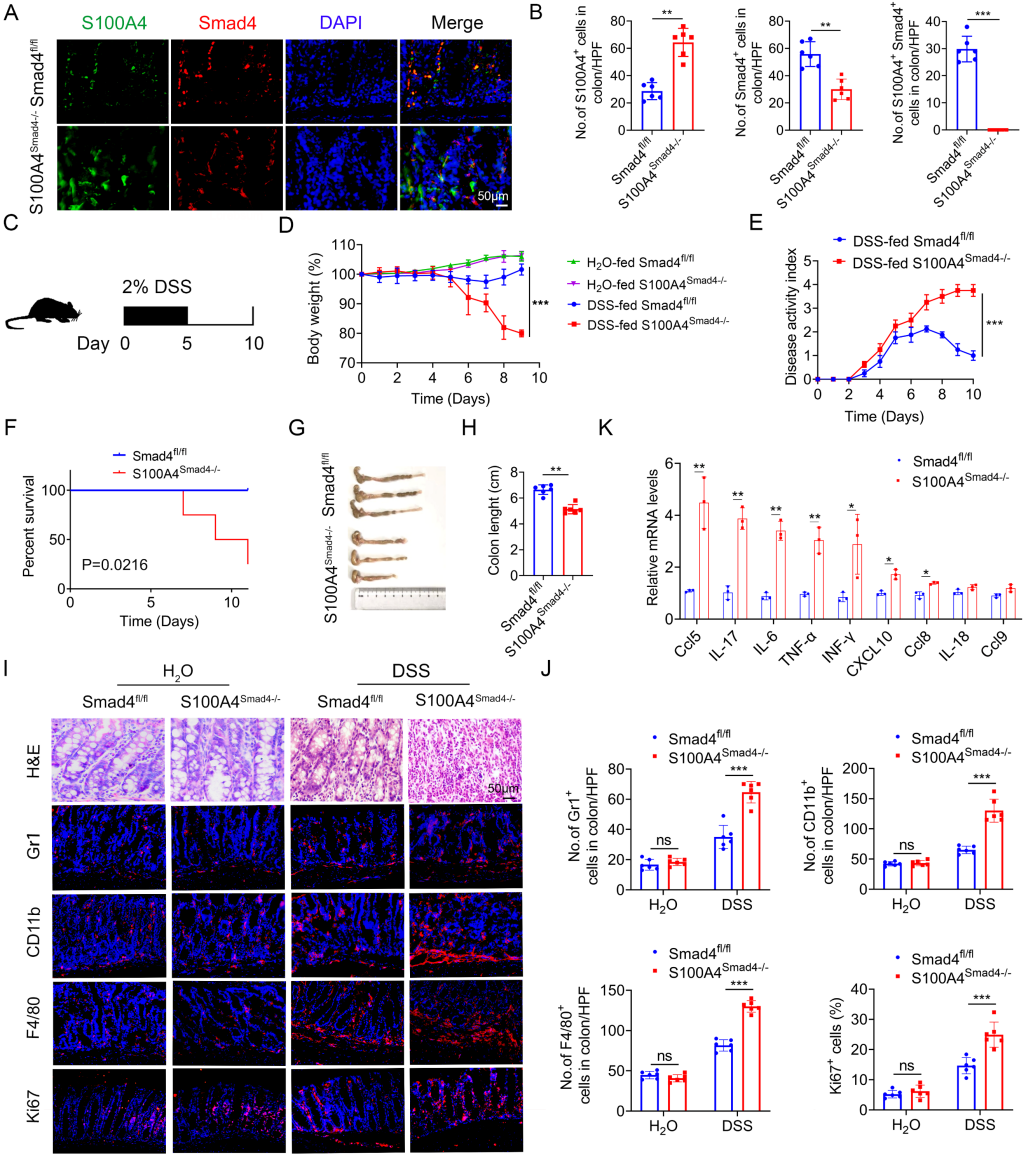
** Smad4 deficiency in S100A4^+^ cells aggravates DSS-induced colitis.** Groups of Smad4^fl/fl^ and S100A4^Smad4-/-^ mice (n = 6 per group) were subjected to the DSS-induced colitis model. The data are representative of three independent experiments. **(A)** Double staining of S100A4 and Smad4 in DSS-induced colon tissues (scale bars: 50 μm) and **(B)** statistical analysis. **P < 0.01, ***P < 0.001. **(C)** Schematic representation of DSS-induced colitis model. **(D)** Body weight changes. ***P < 0.001. **(E)** Disease activity scores. ***P < 0.001. **(F)** Survival curves. *P < 0.05. **(G)** Representative photographs of colon specimens on day 10 following DSS treatment, and **(H)** colon lengths on day 10. **P < 0.01. **(I)** Hematoxylin and eosin (H&E) (Scale bar: 50 μm), Gr1, CD11b, F4/80, and Ki67 staining in colon specimens (Scale bar: 100 μm) and **(J)** statistical analysis. ***P < 0.001. **(K)** Cytokine mRNA expression in colon tissue induced by DSS. *P < 0.05, **P < 0.01.

To determine the role of Smad4 in S100A4^+^ cells, we crossed mice carrying the loxP-flanked Smad4 allele with S100A4-cre mice to achieve Smad4 ablation specifically in S100A4^+^ cells. The resulting S100A4-cre-Smad4^flox/flox^ (S100A4^Smad4-/-^) mice were viable and fertile and did not exhibit any spontaneous intestinal phenotype. The absence of Smad4 in S100A4^+^ cells obtained with S100A4^Smad4-/-^ mice was confirmed via double staining of Smad4 and S100A4 (Figures 2A, B). For the induction of the acute colitis model, 2% DSS in the drinking water was administered to Smad4^fl/fl^ and S100A4^Smad4-/-^ mice for 5 days, followed by 5 days of regular drinking water (Figure 2C). As shown in Figure 2D, S100A4^Smad4-/-^ mice were more susceptible to DSS induction than control mice. Weight loss in S100A4^Smad4-/-^ mice was considerably higher on days 6, 8 and 10. Smad4^fl/fl^ mice began to recover on day 8 following severe body weight loss, whereas S100A4^Smad4-/-^ mice did not show a trend towards recovery, and the body weight loss was further exacerbated on day 10. The disease score in S100A4^Smad4-/-^ mice was considerably higher than Smad4^fl/fl^ mice on days 8 and 10 (Figure 2E). Consequently, S100A4^+^ cell Smad4 knockout significantly increased the mortality of mice following DSS induction. As shown in Figure 2F, S100A4^Smad4-/-^ mice began to die from day 6, reaching 75% mortality on day 11, whereas no deaths occurred in the control mice.

In addition, the colon lengths in S100A4^Smad4-/-^ mice were shorter than those in Smad4^fl/fl^ mice on day 10 (Figures 2G, H). Consistently, histological analysis revealed that S100A4^Smad4-/-^ mice exhibited much more severe colonic damage and more inflammatory cell infiltration on day 10 (Figure 2I). Furthermore, the infiltration of Gr1^+^ neutrophils, CD11b^+^ macrophages, and F4/80^+^ macrophages was considerably higher in S100A4^Smad4-/-^ mice on day 10 (Figure 2J). Moreover, the proportion of proliferating (Ki67^+^) cells in colon tissues was significantly increased in S100A4^Smad4-/-^ mice. There were no differences in hematoxylin and eosin (H&E) staining, inflammatory cell infiltration or proportion of proliferating cells between the colonic tissues of drinking water-treated S100A4^Smad4-/-^ mice and control mice (Figure 2J). In addition, the mRNA levels of proinflammatory cytokines including Ccl5, IL-17, IL-6, TNF-α, IFN-γ, CXCL10, and Ccl8 were all found to be increased in colitis tissues of S100A4^Smad4-/-^ mice compared with those in Smad4^fl/fl^ mice (Figure 2K). These results indicated that specific knockout of Smad4 in S100A4^+^ cells exacerbated colitis by facilitating the recruitment of inflammatory cells and augmenting the inflammatory response.

### Smad4 deletion in S100A4^+^ cells promotes colitis-associated colorectal tumorigenesis

**Figure 3:**
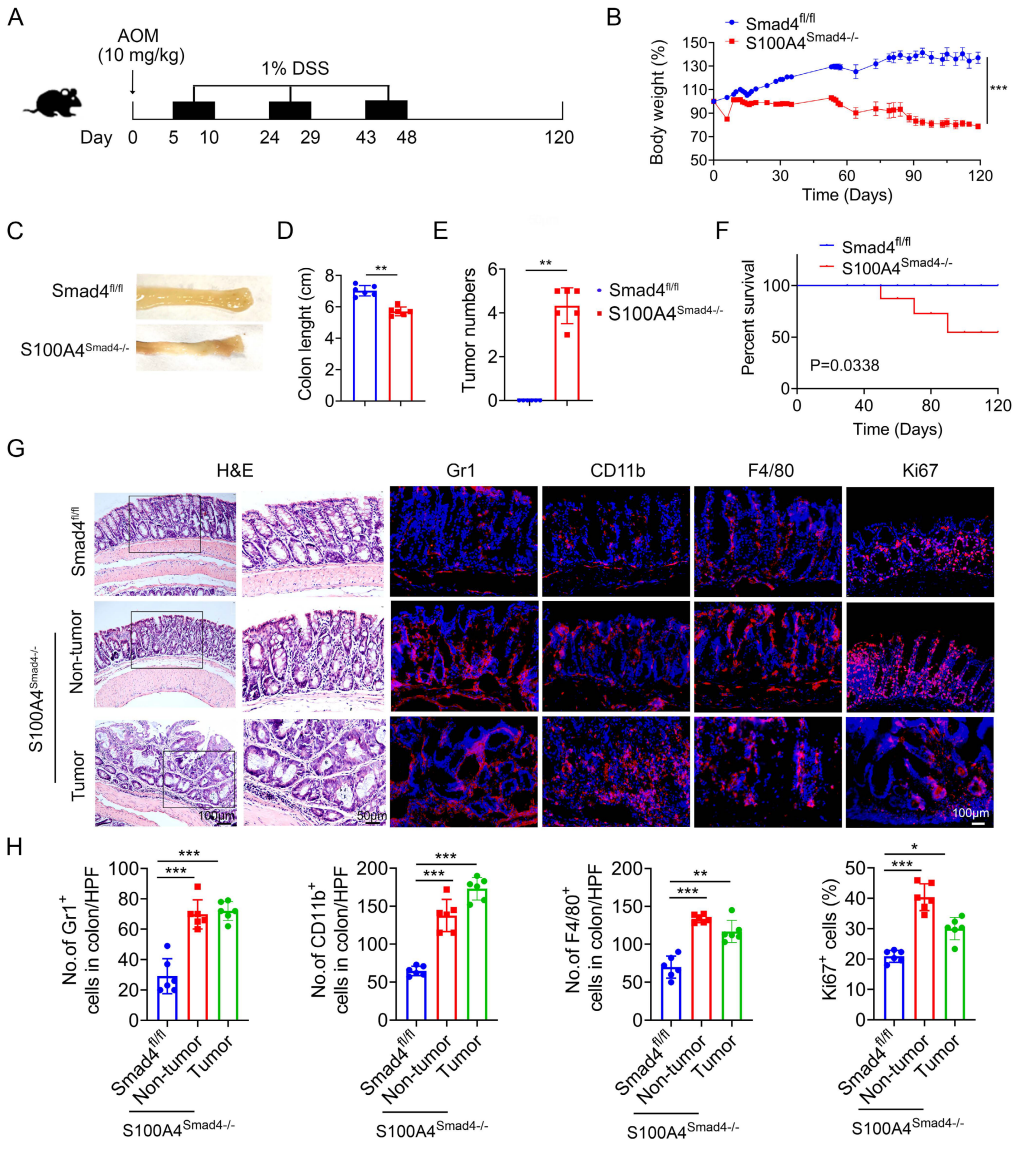
** Smad4 deletion in S100A4^+^ cells promotes colitis-associated colorectal tumorigenesis.** Groups of Smad4^fl/fl^ and S100A4^Smad4-/-^ mice (n = 6 per group) were subjected to the AOM/DSS-induced CAC model. The data are representative of three independent experiments. **(A)** Schematic representation of the AOM/DSS-induced CAC model. **(B)** Body weight changes. ***P < 0.001. **(C)** Representative images of colon specimens on day 120 following AOM/DSS treatment, **(D)** colon length, **(E)** number of tumors per mouse. **P < 0.01, ***P < 0.001. **(F)** The survival rates. *P < 0.05. **(G)** H&E (Scale bar: 50 μm), Gr1, CD11b, F4/80, Ki67staining of colon specimens (Scale bar: 100 μm), and **(H)** statistical analysis. *P < 0.05, **P < 0.01, ***P < 0.001.

Chronic inflammation is a key risk factor for CRC in patients with IBD. The risk of developing CRC increases with a longer duration of colitis 27. To further determine the role of Smad4 in S100A4^+^ cells in CAC, S100A4^Smad4-/-^ and control mice were subjected to AOM/DSS. After a single intraperitoneal injection of AOM followed by three cycles of 1% DSS administration in their drinking water, and monitored for 120 days (Figure 3A). Compared with Smad4^fl/fl^ mice, S100A4^Smad4-/-^ mice exhibited greater weight loss during the first cycle of DSS treatment (Figure 3B) and decreased colon length at the end of the protocol (Figures 3C, D), indicating that S100A4^Smad4-/-^ mice had a stronger inflammatory response. Consistently, S100A4^Smad4-/-^ mice were more susceptible to tumor development (Figure 3E). Because the DSS concentration was very low, at only 1%, all of the control mice were tumor-free at Day 120, whereas all of the S100A4^Smad4-/-^ mice developed tumors. The mortality was significantly increased in S100A4^Smad4-/-^ mice, as 50% of the mice died on day 90, whereas no deaths occurred in the control mice (Figure 3F). Pathological analysis and proliferating cell staining showed that tumor growth and progression were enhanced in the S100A4^Smad4-/-^ mice (Figures 3G, H). Additionally, the number of CD11b^+^, F4/80^+^, and Gr1^+^ cells was significantly increased. These results suggest that Smad4 depletion in S100A4^+^ cells promotes CAC tumorigenesis.

### Smad4-specific deletion in S100A4^+^ cells promotes macrophage M2 polarization

**Figure 4:**
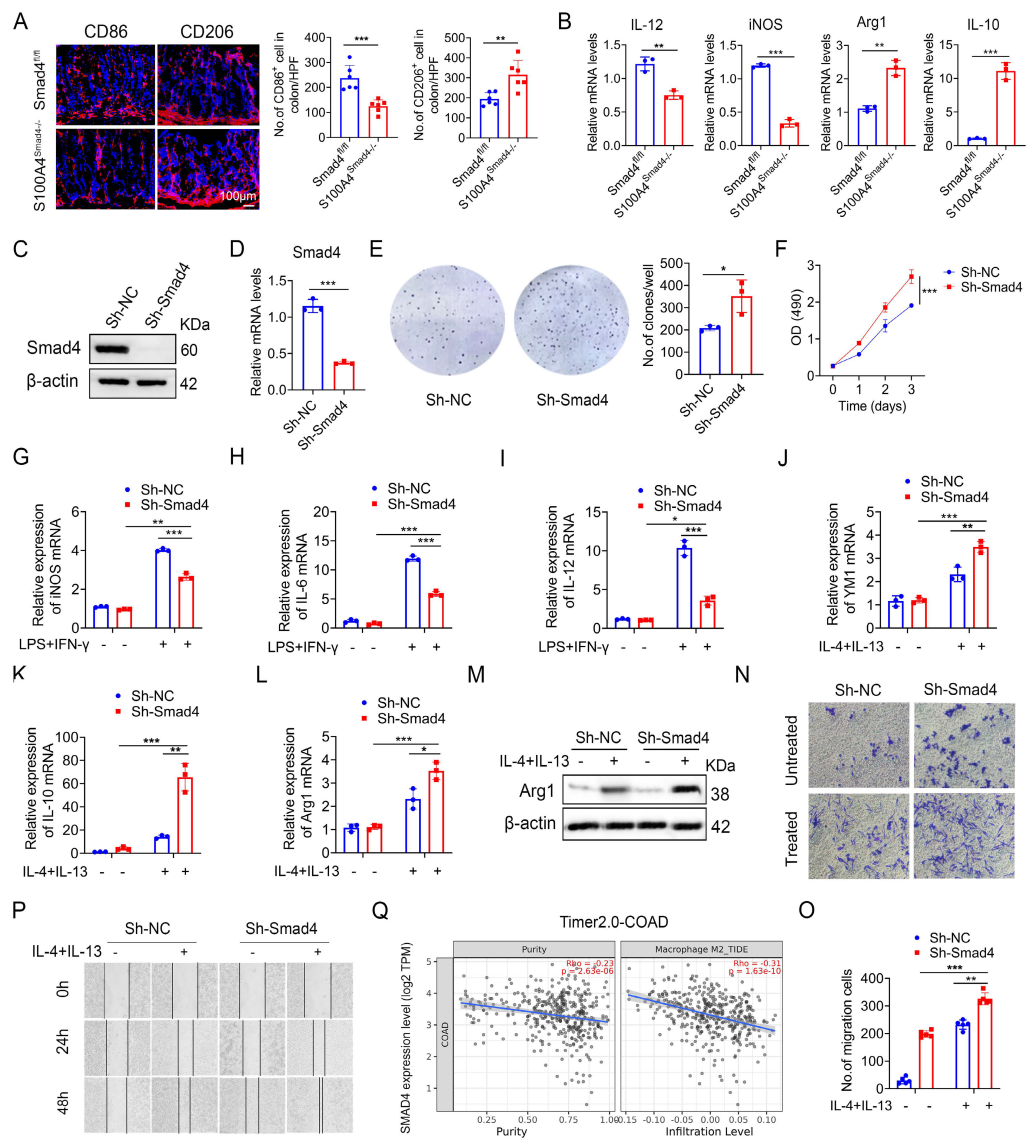
** Smad4-specific deletion in S100A4^+^ cells promotes macrophage M2 polarization. (A)** Representative immunofluorescence staining for CD86 and CD206 in AOM/DSS-induced colon tissues (Scale bar: 100 μm) and statistical analysis. *P < 0.05, **P < 0.01. **(B)** The mRNA levels of IL-12, iNOS, Arg1, and IL-10 in AOM/DSS-induced colon tissues. **P < 0.01, ***P <0.001, **(C)** Western blot, and **(D)** qPCR results verified Smad4 knockdown in Raw264.7 cells. ***P < 0.001. **(E)** Representative images of the clonal formation of Raw264.7 cells and statistical analysis. *P < 0.05. **(F)** Cell viability was measured by methyl thiazole tetrazolium (MTT) assay in Raw264.7 cells. ***P < 0.001. **(G-I)** iNOS, IL-6, and IL-12 mRNA expression in 100 ng/mL LPS and 20 ng/mL IFN-γ stimulation of Raw264.7 cells. *P < 0.05, **P < 0.01, ***P < 0.001. **(J-L)** YM1, IL-10, and Arg1 mRNA expression in 20 ng/mL IL-4 and 20 ng/mL IL-13 stimulation of Raw264.7 cells. *P < 0.05, **P < 0.01, ***P < 0.001. **(M)** Arg1 protein expression in Raw264.7 cells. **(N-O)** The chemotaxis of Raw264.7 cells was examined by transwell assay. **P < 0.01, ***P < 0.001. **(P)** Scratch assay of Raw264.7 cells. **(Q)** Spearman correlation between Smad4 and M2 macrophage in colorectal cancer analyzed by the Time2.0 database. The data are representative of at least three independent experiments.

Macrophages play an important role both in inflammatory response and tumor immunity. They are polarized into the M1 and M2 phenotypes under different conditions 28. We analyzed the number of CD86^+^ (an M1 marker) and CD206^+^ (an M2 marker) macrophages in colon tumor tissues and revealed that S100A4^Smad4-/-^ mice had reduced M1 phenotype and enhanced M2 phenotype (Figure 4A). Similarly, Smad4 deletion in S100A4^+^ cells down-regulated the expression of M1-like factors such as iNOS and IL-12 and up-regulated the expression of M2-related genes such as Arg1 and IL-10 in CRC tumor tissues (Figure 4B).

Previous studies have shown that most of the S100A4^+^ cells were macrophages in colon and tumor tissues. To further examine the role of Smad4 in regulating macrophage polarization, we knocked down Smad4 in Raw264.7 cells using lentivirus infection to establish a Smad4 knockdown (Sh-Smad4) Raw264.7 cell line. Successful knockdown was confirmed via Western blot analysis (Figure 4C) and real-time quantitative PCR (qPCR) (Figure 4D). Smad4 knockdown promoted the proliferation of Raw264.7 cells, as determined via clonal formation (Figure 4E) and methyl thiazole tetrazolium (MTT) assays (Figure 4F). Moreover, IL-10 and CXCL10 secretion was increased following Smad4 knockdown in Raw264.7 cells treated with LPS (Figure S1A-E).

We next examined the specific role of Smad4 in macrophage polarization *in vitro*. Raw264.7 cells were cultured with LPS and IFN-γ for 24 h to induce M1 polarization or treated with IL-4 and IL-13 for 48 h to induce M2 polarization. Following the M1 polarization induction, iNOS, IL-6, and IL-12 mRNA levels in Sh-smad4 Raw264.7 cells were significantly down-regulated (Figures 4G, I). Conversely, Sh-Smad4 Raw264.7 cells induced M2 polarization via IL-4 and IL-13, and the results showed increased expression of M2 markers such as IL-10, Arg1, and YM1 (Figures 4J, L). Western blot analysis further confirmed that Smad4 knockdown promoted macrophage M2 polarization by regulating arginase expression (M2 marker) (Figure 4M). Interestingly, Smad4 knockdown promoted programmed cell death protein 1 (PD-1) expression in Raw264 cells, as determined via flow cytometry analysis (Figures S2A-B). This difference was more obvious following the M2 polarization induction by IL-4 and IL-13 (Figure S2C). These results suggest that Smad4 in macrophages is associated with anti-tumor immunity.

Next, the effect of Smad4 in macrophages on the migration ability was examined. Transwell migration (Figures 4N, O) and cell scratch assays (Figure 4P) revealed that Smad4 knockdown promotes the migration ability of macrophages. This trend is more pronounced in Raw264.7 cells following IL-4 and IL-13 stimulation. Furthermore, Smad4 expression was negatively correlated with CRC and M2 macrophages analyzed by the Time2.0 database (Figure 4Q). Taken together, these findings suggest that Smad4 deficiency promotes the transformation of macrophages towards M2 polarization and enhances the chemotactic ability of macrophages.

### Smad4 deficiency in S100A4^+^ cells facilitates FA metabolism

**Figure 5:**
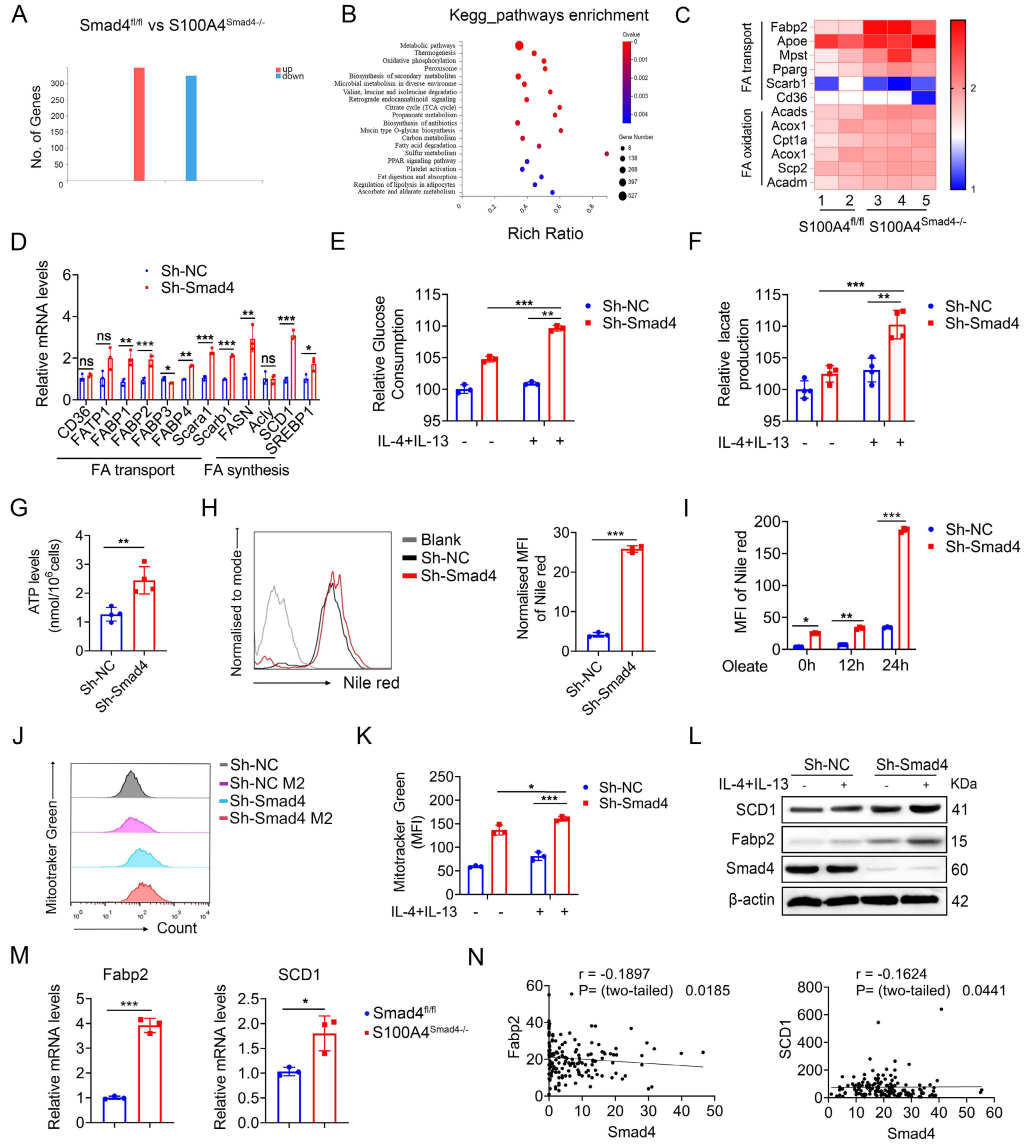
** Smad4 depletion increases macrophagic capability in the usage of exogenous Fatty acid (FA).** RNA sequencing analysis of differentially expressed genes (DEGs) between DSS-induced colitis tissues from Smad4^fl/fl^ and S100A4^Smad4-/-^ mice*.* **(A)** Histogram of DEGs, the threshold is P < 0.05, and the absolute value of log2 Ratio ≥ 1. **(B)** Gene Ontology (GO) analysis of DEGs. **(C)** Heatmap of the most significant DEGs related to FA metabolism. **(D)** After 20 ng/mL IL-4 and 20 ng/mL IL-13 stimulation of Raw264.7 cells with 48 hours, the mRNA changes of FA transport and synthesis-related genes were detected. *P < 0.05, **P < 0.01, ***P < 0.001. **(E-F)** After 20 ng/mL IL-4 and 20 ng/mL IL-13 stimulation of Raw264.7 cells with 48 hours, glucose consumption and lactate production capacity of Raw264.7 cells were detected. **P < 0.01, ***P < 0.001. **(G)** ATP levels in Raw264.7 cells. **P < 0.01. **(H)** After staining with Nile Red, lipid droplets in Raw264.7 cells of Sh-Smad4 and Sh-NC were detected by flow cytometry. ***P < 0.001. **(I)** Oleate (1×) of free FA was added into the culture system. After Nile Red staining at different times of 0h, 12h, and 24h, intracellular lipid droplets of Raw264.7 cells were detected by flow cytometry. *P < 0.05, **P < 0.01, ***P < 0.001. **(J)** Flow cytometry analysis of MFI of Mitotracker Green of Raw264.7 cells and **(K)** statistical analysis. *P < 0.05, ***P < 0.001. **(L)** Protein levels of stearoyl-CoA desaturase-1 (SCD1) and FA binding protein 2 (Fabp2) in Raw264.7 cells were detected after IL-4 (20 ng/mL), and IL-13 (20 ng/mL) treatment. **(M)** The mRNA levels of Fabp2 and SCD1 in colon tissues induced with AOM/DSS. *P < 0.05, ***P < 0.001. **(N)** Correlations between the expression of Fabp2 or SCD1 and Smad4 in patient-derived tissues with colorectal cancer (n = 154), P < 0.05 were considered statistically significant. MFI: mean fluorescence intensity. The data are representative of at least three independent experiments.

To gain insight into Smad4 function during the alternative polarization of TAMs, RNA sequencing was performed on DSS-induced colitis tissues obtained from both Smad4^fl/fl^ and S100A4^Smad4-/-^ mice. A total of 662 differentially expressed genes (DEGs) were identified, including 336 up-regulated and 326 down-regulated genes (Figure 5A; padj < 0.05; absolute value of log2 ratio ≥ 1). A Kyoto Encyclopedia of Genes and Genomes (KEGG) analysis (Figure S3A) and Gene Ontology (GO) (Figure 5B) revealed that significant changes occurred in processes related to FA metabolism, such as FA degradation, fat digestion and absorption in S100A4^Smad4-/-^ mice compared with Smad4^fl/fl^ mice. Heat maps of the differential genes showed that expressions of FA-binding protein 2 (Fabp2), Hmgcs2, and Cldn15 in S100A4^Smad4-/-^ mice were significantly up-regulated and most of them were related to metabolism (Figures S3B-C). Subsequently, a focused examination of lipid metabolism in a heat map (Figure 5C) and statistical analysis (Figure S3D) revealed the significant upregulation of Fabp2. Collectively, these findings suggest that Smad4 knockdown in S100A4^+^ cells affects lipid metabolism, and Fabp2 may be a downstream target of Smad4 action.

### Smad4 deficiency increases macrophagic capability in the usage of exogenous FAs

To clarify the specific role of Smad4 in FA metabolism, genes involved in FA synthesis and transport were examined. Sh-Smad4 Raw 264.7 significantly up-regulated the expression of most genes involved in FA synthesis and transport (Figure 5D), suggesting that Smad4 deletion in Raw264.7 cells promoted FA metabolism. Metabolic transfer between glycolysis and mitochondria is closely associated with the phenotype of macrophages, and the end-products of glycolysis and FA metabolism are used by mitochondria in the TCA cycle 29. Therefore, we examined the role of Smad4 in regulating mitochondrial activity. Smad4 knockdown in Raw264.7 promoted glucose uptake (Figure 5E) and lactate production following IL-4 and IL-13 induction (Figure 5F). Moreover, the ATP production of Sh-Smad4 Raw 264.7 cells was also significantly increased (Figure 5G). Flow cytometry results confirmed that the mitochondrial membrane potential of Sh-Smad4 cells induced into the M2 type was enhanced (Figures 5I, J). These findings suggest that Smad4 knockdown in macrophages promotes metabolic reprogramming in mitochondria.

Intracellular lipid droplets provide a stable source of FAs for TAMs, and control macrophage polarization towards an M2-like phenotype 30. To determine the effects of Smad4 knockdown on FA uptake and lipid droplets that form in Raw264.7 cells, we evaluated the lipid droplets in Raw264.7 cells under normal culture conditions. Lipid droplet content of Sh-Smad4 Raw264.7 increased significantly (Figure 5H). Moreover, Smad4 knockdown promoted FA uptake and lipid synthesis in macrophages, particularly in oleate culture for 24 h (Figure 5I). Following IL-4 and IL-13 exposure, Smad4 knockdown in macrophages significantly promoted stearoyl-CoA desaturase-1 (SCD1) (a key gene in FA metabolism) and Fabp2 expression as determined by western blot analysis (Figure 5L). Similar results were also observed in AOM/DSS-induced colon tissues by qPCR (Figure 5M). Transcriptome sequencing analysis of patient colon cancer tissues revealed that Smad4 expression was negatively correlated with Fabp2 and SCD1 expression (Figure 5N). These results indicate that Smad4 deficiency promotes FA uptake and metabolic reprogramming in macrophages.

### Smad4 knockdown facilitates macrophage polarization and lipid metabolism through the FABP2/STAT6 pathway

**Figure 6:**
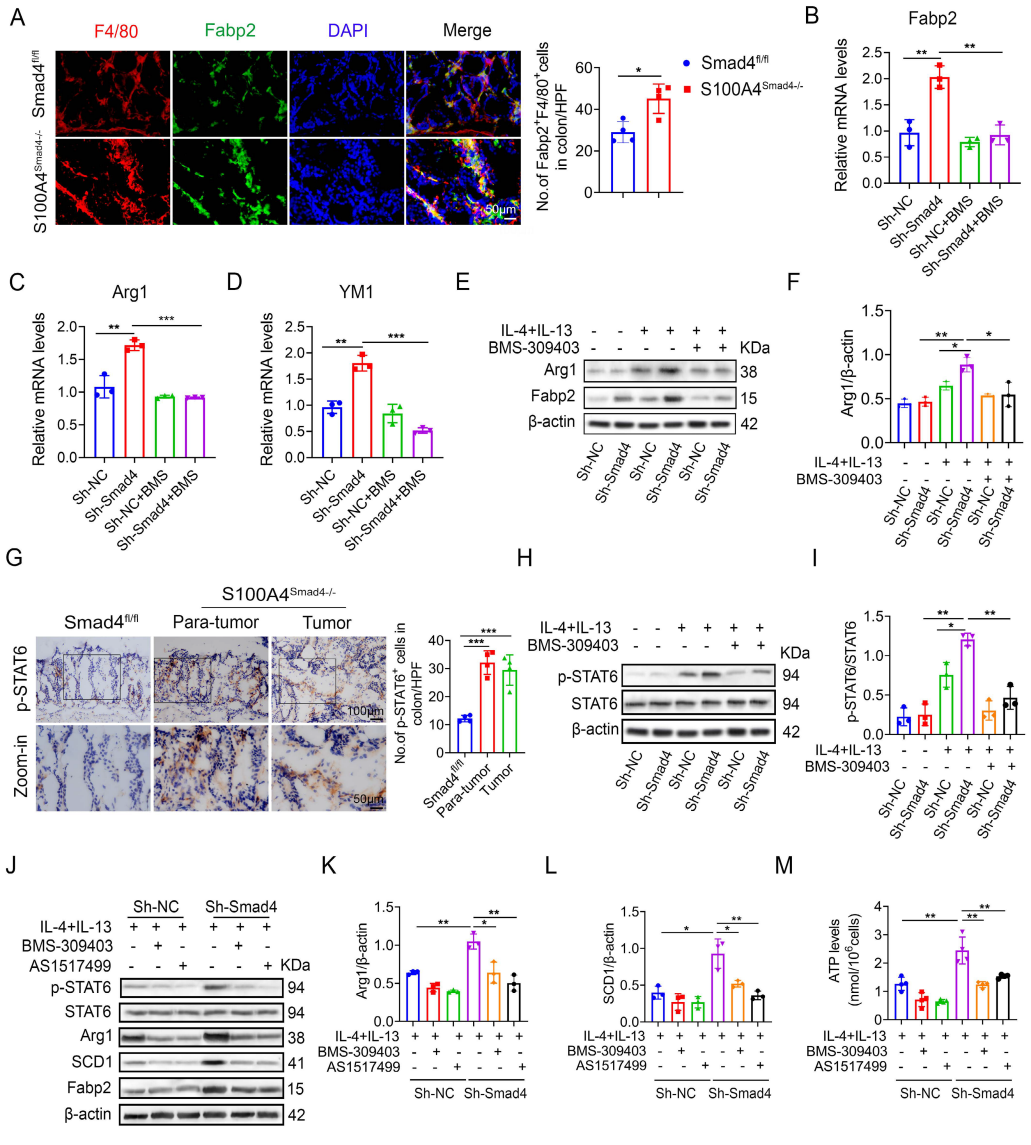
** Smad4 knockdown facilitates macrophage polarization and lipid metabolism through the FABP2/STAT6 pathway. (A)** Double staining of F4/80 and Fabp2 in DSS-induced colon tissues (Scale bar: 50 μm) and statistical analysis. *P < 0.05 **(B-D)** mRNA levels of **(B)** Fabp2, **(C)** Arg1, and **(D)** YM1 in Raw 264.7 cells treated with IL-4, IL-13, and BMS-309403 (a Fabp inhibitor) (40 uM). **P < 0.01, ***P < 0.001. **(E-F)** Protein levels of Arg1 and Fabp2 in Raw264.7 cells were detected after IL-4, IL-13, and BMS-309403 treatment. *P < 0.05, **P < 0.01. **(G)** Representative staining of p-STAT6 in colon specimens (scale bars: 50 μm) and statistical analysis. ***P < 0.001. **(H)** Protein levels of p-STAT6 and STAT6 in Raw264.7 cells were detected after IL-4, IL-13, and BMS-309403 treatment and **(I)** statistical analysis. *P < 0.05, **P < 0.01. b Raw264.7 cells treated with IL-4, IL-13, BMS-309403, and AS1514799 (a STAT6 inhibitor), the protein levels of Arg1 **(K)** and SCD1 **(L)** were detected. *P < 0.05, **P < 0.01. **(M)** ATP levels in Raw264.7 cells. **P < 0.01. The data are representative of at least three independent experiments.

Sequencing results revealed that the Fabp2 expression in DSS-induced colon tissues of S100A4^Smad4-/-^ mice was higher compared with the Smad4^fl/fl^ group. Fabp2 is a key gene involved in lipid transport and metabolism 31. The change in the lipid metabolism of macrophages results in the transformation of the polarization phenotype 32. Our results demonstrate that Smad4 knockdown in S100A4^+^ cells promotes macrophage M2 polarization. The number of Fabp2^+^ F4/80^+^ cells in S100A4^Smad4-/-^ mice increased significantly (Figure 6A). The mRNA and protein levels of Fabp2 in Sh-Smad4 macrophages increased markedly following M2-induction (Figures 6B, E). Treatment of Raw264.7 cells with BMS-309403 (BMS, a Fabp inhibitor) attenuated IL-4 and IL-13-induced Arg1 (an M2 marker) expression in the Sh-Smad4 group (Figures 6C, F), suggesting that Smad4 knockdown promotes the transformation of macrophages towards M2 polarization by targeting Fabp2 expression.

STAT6 pathway is an important signaling pathway involved in the regulation of macrophage polarization 33. In AOM/DSS-induced colon tissues, p-STAT6 expression was higher in S100A4^Smad4-/-^ mice than in control mice (Figure 6G). Smad4 knockdown promoted IL-4 and IL-13-induced p-STAT6 expression in Raw264.7 cells; however, BMS-309403 reversed this effect as determined by Western blot (Figure 6H, I), suggesting that Smad4 deficiency promotes STAT6 phosphorylation by increasing Fabp2 expression.

To further confirm whether Smad4 affects macrophage metabolism and phenotype via the Fabp2/STAT6 pathway, the Raw264.7 cells were treated with BMS-309403 and AS1517499 (a STAT6 inhibitor) (Figure 6J). Western blot analysis revealed that BMS-309403 and AS1517499 reversed the increase in Arg1 (Figure 6K) and SCD1 (Figure 6L) expression in Sh-Smad4 Raw264.7 cells with M2 induction. Furthermore, Smad4 knockdown promoted ATP production in Raw264.7 cells induced by IL-4 and IL-13. In contrast, BMS-309403 and AS1517499 attenuated the ATP levels (Figure 6M). These results demonstrate that Smad4 deficiency promotes M2 polarization and FA metabolism through the activation of Fabp2 and STAT6 signals in macrophages.

### Smad4 deficiency in myeloid cells promotes MC38 tumor growth via Fabp2-dependent M2 polarization

**Figure 7:**
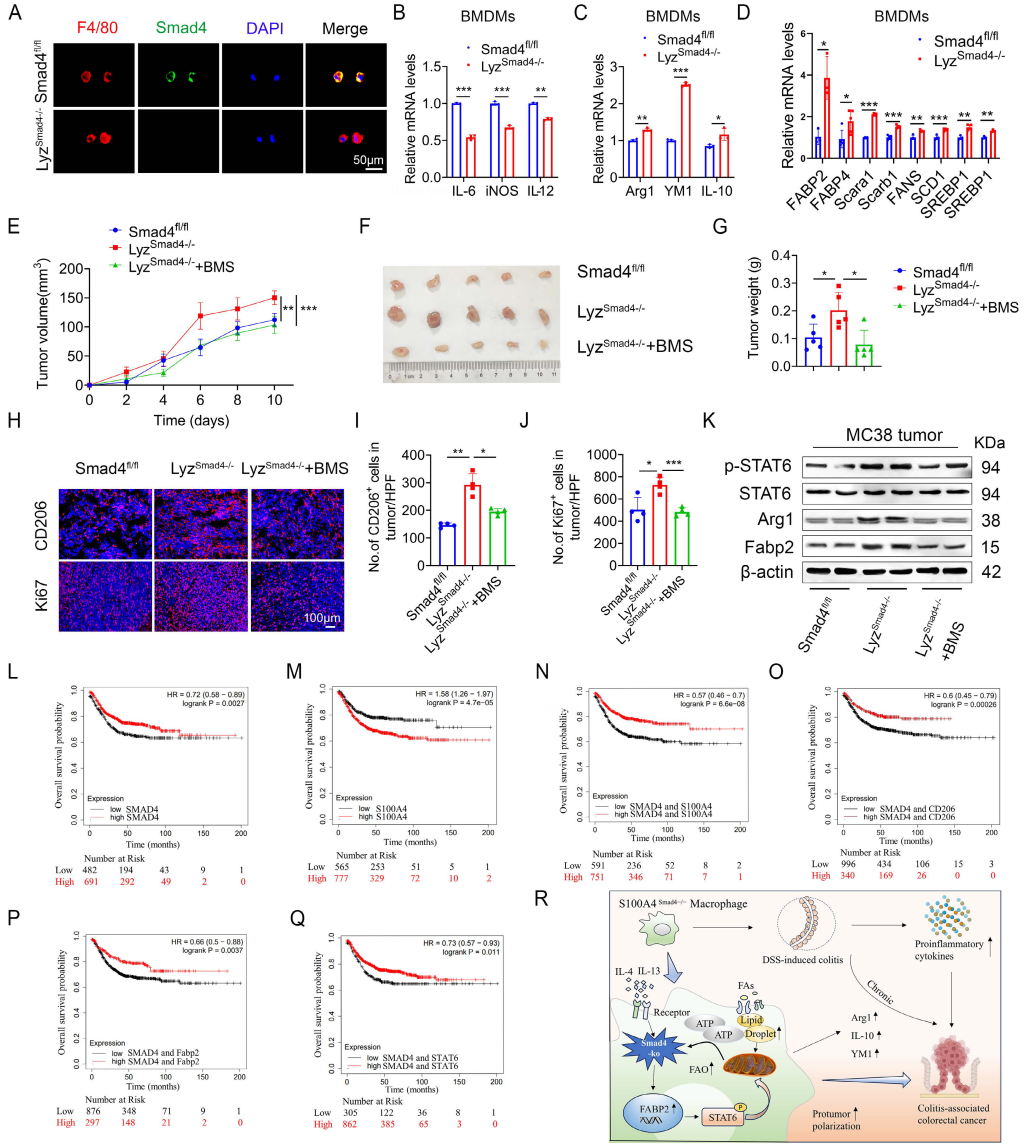
** Myeloid-specific Smad4 deficiency promoted MC38 tumor growth via Fabp2-dependent M2 polarization and Smad4 is associated with longer survival of patients with CRC. (A)** Double staining of F4/80 and Smad4 in Bone marrow-derived macrophages (BMDMs) from Smad4^fl/fl^ and Lyz^Smad4-/-^ mice (50 μm) and statistical analysis. **P < 0.01, ***P < 0.001. **(B)** Smad4^fl/fl^ and Lyz^Smad4-/-^ BMDMs were treated with IFN-γ (20 ng/mL) in combination with LPS (100 ng/mL), IL-6, iNOS, and TNF-α mRNA expression was analyzed. **P < 0.01, ***P < 0.001. **(C)** BMDMs were treated with IL-4 (20 ng/mL) and IL-13 (20 ng/mL), Arg1, YM1, and IL-10 mRNA expression was analyzed. *P < 0.05, **P < 0.01, ***P < 0.001. **(D)** After 20 ng/mL IL-4 and 20 ng/mL IL-13 stimulation of BMDMs with 48 hours, the mRNA changes of FA metabolism-related genes were detected. *P < 0.05, **P < 0.01, ***P < 0.001. **(E-G)** 7×10^5^ MC38 tumor cells were inoculated into the flank of Smad4^fl/fl^ and Lyz^Smad4-/-^ mice. Groups of Lyz^Smad4-/-^+BMS (n = 4 per group) mice were injected with BMS-309403 (10 mg/kg) once every two days, groups of Smad4^fl/fl^ and Lyz^Smad4-/-^ (n = 4 per group) mice were injected with PBS at the same time. **(E)** Growth curves of tumor volume. **P < 0.01, ***P < 0.001. **(F)** Representative tumor images, and tumor burdens **(G)** were shown. *P<0.05. **(H-J)** Ki67 and CD206 staining in tumor tissues and statistical analysis (Scale bar: 100 μm). *P < 0.05, **P < 0.01, ***P < 0.001. **(K)** Protein levels of p-STAT6, STAT6, Fabp2, and Arg1in tumor tissues. **(L-Q)** Kaplan-Meier curve for survival in the Kaplan Meier-plotter dataset with combined Smad4/S100A4/CD206/Fabp2/STAT6 gene expression in patients with colorectal cancers bifurcated at the median expression level into high vs low. **(R)** Mechanism diagram of Smad4 signaling in S100A4+ macrophages acting model in colitis and colon cancer. The data are representative of at least three independent experiments.

To further determine the role of Smad4 in macrophages, mice with a conditional knockout of Smad4 in myeloid cells (Lyz^Smad4-/-^ mice) were generated by crossing Smad4^flox/flox^ and Lyz-Cre mice. The absence of Smad4 in myeloid cells from the Lyz^Smad4-/-^ mice was confirmed by double staining of F4/80 and Smad4 (Figure 7A). Smad4 ablation in BMDMs was confirmed by qPCR, and the cells were polarized to M1 and M2 macrophages with LPS/IFN-γ and IL-4/IL-13, respectively. Smad4 ablation inhibited the expression of IL-6, iNOS, and TNF-α (M1 marker) following LPS/IFN-γ stimulation (Figure 7B). Conversely, Smad4 deletion increased Arg1, YM1, and IL-10 (M2 marker) mRNA expression following IL-4/IL-13 stimulation (Figure 7C). Furthermore, myeloid cell-specific Smad4 deficiency promoted the mRNA expression of genes related to lipid metabolism; this finding was consistent with that observed in Raw264.7 cells (Figure 7D). These results indicate that myeloid cell-specific Smad4 deletion promotes the M2 polarization and lipid metabolism in macrophages, consistent with the results observed in Raw264.7 cells.

To further explore the role of Smad4 in macrophages during colon cancer progression, MC38 cells were subcutaneously inoculated into Lyz^Smad4-/-^ mice and Smad4^fl/fl^ mice. To evaluate the therapeutic effects, Lyz^Smad4-/-^ mice received an intraperitoneal injection of Fabp inhibitor (BMS309403). Tumor growth was assessed for two weeks. Smad4 knockout in myeloid cells significantly promoted tumor growth. BMS309403 effectively reversed the accelerated tumor growth caused by myeloid cell-specific Smad4 deletion (Figures 7E-G). Moreover, IF staining revealed that the number of CD206^+^ cells and Ki67^+^ cells was remarkably increased in Lyz^Smad4-/-^ mice. BMS309404 treatment significantly decreased the number of CD206^+^ cells and Ki67^+^ cells (Figures 7H-J). Furthermore, western blot analysis of tumor tissues confirmed that myeloid cell-specific Smad4 ablation promoted STAT6 phosphorylation, whereas blocking Fabp2 reversed this phenomenon (Figure 7K). In addition, the supernatant from IL-4/IL-13-induced Sh-Smad4 Raw264.7 cells enhanced the proliferative activity of MC38 cells, and the proliferative effect was reversed by BMS309403 (Figure S4A). Taken together, Smad4 deficiency in macrophages promotes M2 polarization via Fabp2 expression in transplanted tumors. Furthermore, using Fabp inhibitor to block Fabp2 results in an anti-tumor effect.

### Smad4 expression is associated with prolonged survival in patients with CRC

To extrapolate our results to humans, we used the Kaplan Meier plotter database. The database was established using on-chip gene expression and the data was downloaded from the gene expression Omnibus. High Smad4 expression predicted high overall survival in CRC patients (Figure 7L), whereas high S100A4 expression predicted low overall survival in CRC patients (Figure 7M). Next, we divided the patients into S100A4^Low^ and S100A4^Low^/CD206^Low^/Fabp2^Low^/STAT6^Low^, S100A4^High^ and S100A4^High^/CD206^High^/Fabp2^High^/STAT6^High^ each in the two groups. Kaplan-Meier survival curve revealed that the overall survival rate of Smad4 and S100A4/CD206/Fabp2/STAT6 with comprehensive low expression was reduced significantly (Figures 7N-Q). The results indicated that Smad4 expression is positively associated with a favorable prognosis of colon cancer and Smad4 may serve as a strong predictor of survival in patients with CRC.

## Discussion

The role of Smad4 in S100A4^+^ cells in CAC was examined. We found that S100A4^+^ cell-specific Smad4 deletion aggravated DSS-induced colitis and enhanced AOM/DSS-induced colitis-associated tumorigenesis. Moreover, Smad4 deficiency in S100A4^+^ cells promoted colonic macrophage infiltration and M2 polarization. Smad4 deletion resulted in increased FA uptake and transport in macrophages and promoted macrophage M2 polarization through the activation of Fabp2 and the STAT6 pathway (Figure 7R).

Smad4 acts as a tumor suppressor in CRC; however, the role of Smad4-mediated signaling in the immune microenvironment of CRC remains controversial Smad4 deletion in human CRC cells resulted in the recruitment of more neutrophils through the CXCL1/8-CXCR2 axis to promote CRC progression 34. Additionally, Smad4 deficiency in tumor epithelium recruits CCR1-expressing immature myeloid cells to promote tumor invasion at the early stages of intestinal adenocarcinomas 35. Smad4 knockdown in human CRC cell lines recruits CCR1^+^ myeloid cells, and facilitates liver metastasis in tumor-bearing mice by upregulating the expression of CCL5 36. Smad4 deletion in epithelial cells aggravates DSS-induced colitis and AOM/DSS induced-colon tumorigenesis 37. Moreover, Smad4 deficiency in T cells increases the expression of IFN-γ and promotes CAC 38. Similarly, our results supported this conclusion and demonstrated that S100A4^+^ cell-specific Smad4 ablation aggravated colitis and CRC.

Immunosuppressive cell infiltration and cytokine secretion in chronic inflammation contribute to tumor development 39. In this study, we observed increased macrophage infiltration as well as CCL5, CCL8, and CCL9 expression in AOM/DSS-induced CAC in S100A4^Smad4-/-^ mice, consistent with the pathological changes observed during the inflammation-cancer transition. Our results revealed that S100A4^+^ cell-specific Smad4 deficiency aggravates the transformation of colitis into CRC by promoting the infiltration of macrophages and inflammatory factors.

CRC is now recognized as a chronic progressive disease closely associated with metabolic syndrome. Metabolic reprogramming of macrophages and tumor cells provides energy for their growth and proliferation 40. Lipid metabolism is closely related to the phenotypic transformation of macrophages 41. Additionally, FA oxidation (FAO) is an important factor in the polarization of M2 macrophages, and enhanced FAO facilitates the transformation of M1 macrophages into M2 macrophages 42. We demonstrated that Smad4 deletion in Raw264.7 cells promotes FA uptake and oxidation, and inhibits the expression of FA-binding protein reversed macrophage M2 polarization, which highlights that Smad4 deficiency in macrophages facilitates the macrophage transformation from the M1 to M2 phenotype, which is dependent on increased lipid metabolism.

Fabp2 (also known as I-FABP), belongs to the FA-binding protein family, which participates in FA metabolism 43 and nonalcoholic fatty liver disease development 44. When the intestinal epithelial structure is destroyed, Fabp2 is released into the circulation and involved in metabolic regulation 45. Consistent with this finding, RNA-sequencing analysis revealed that Smad4 deletion in S100A4^+^ cells promoted the lipid metabolism and expression of Fabp2. Subsequent studies have demonstrated that suppressing Fabp2 expression weakened macrophage M2 polarization and lipid metabolism following macrophage-specific Smad4 depletion, and effectively reversed the accelerated MC38 tumor growth caused by myeloid cell-specific Smad4 deletion. These results indicated the pro-tumoral effects of Fabp2-mediated macrophage metabolism induced by S100A4^+^ macrophage-specific Smad4 deficiency in CRC.

STAT6 is involved in macrophage M2 polarization 46 and plays an important role in tumorigenesis and malignant transformation 47. We demonstrated increased activation of STAT6 in S1004^Smad4-/-^ mice induced by AOM/DSS. Furthermore, STAT6 is involved in the regulation of FA metabolism and mitochondrial activity 48. Suppression of Fabp2 expression inhibits the phosphorylation of STAT6, which in turn reverses gene expression of M2 polarization and lipid metabolism. This indicates that STAT6 is the major effector of the Smad4/Fabp2 pathway and reinforces the FA uptake in macrophages. However, it remains unclear whether Smad4-specific deletion also affects other pathways.

Taken together, our findings revealed the cell-specific role of Smad4 in colitis and CAC. S100A4^+^ macrophage-specific Smad4 deletion enhances colitis-associated tumorigenesis by promoting macrophage lipid metabolism-dependent M2 polarization, thereby revealing Smad4 in S1000A4^+^ macrophages as a potential prognostic marker for CRC patients.

## Methods

### Cell lines and mice

The Raw264.7 and L929 cell lines were obtained from the American Type Culture Collection (ATCC; Manassas, VA, USA), and the MC38 cell lines were purchased from the iCell (Bioscience Inc, Shanghai). The cells were cultured in Dulbecco's modified Eagle's medium (DMEM) with 10% fetal bovine serum (FBS) at 37^o^C with 5% CO_2_.

S100A4-Cre, Lyz-Cre, and Smad4^ flox/flox^ (Smad4^fl/fl^) mice on a C57BL/6 background were purchased from Jackson Laboratory (Bar Harbor, ME, USA). Mice with a conditional Smad4 knockout in S100A4-expressing cells (S100A4^Smad4-/-^) were generated by crossing Smad4^fl/fl^ and S100A4-Cre mice. Mice with a conditional knockout of Smad4 in myeloid cells (Lyz^Smad4-/-^ mice) were generated by crossing Smad4^fl/fl^ and Lyz-Cre mice. The Smad4^fl/fl^ mice as controls and all mice were bred under specific pathogen-free conditions in the animal facilities at the Institute of Biophysics, Chinese Academy of Sciences.

### Induction of colitis and CAC

Colitis was induced in 8-10-week-old male mice with 2.0% (w/v) Dextran sodium sulfate (DSS) (MW: 36,000-50,000 D; MP Biomedicals, Canada) dissolved in sterile, distilled water for 5 days, followed by 5 days of normal water.

For CAC, the male mice were injected intraperitoneally with 10 mg/kg Azoxymethane (AOM) (Sigma, St Louis, MO). On day 6, 1% DSS was given in drinking water for 5 days, then drinking water for 2 weeks. Repeated the cycle for 3 times, and the mice were killed on the 120th day.

### Histopathology and immunofluorescence staining analysis

The obtained colon was rolled into a so-called ''Swiss roll'' and fixed with 4% PFA. Paraffin-embedded sections were cut into 5 μm and H&E staining was performed.

For immunofluorescence (IF), frozen colon sections were stained with anti-CD8, anti-CD4, anti-F4/80, anti-Gr1, and anti-CD11b (BD Pharmingen, San Diego, CA). Colon sections were incubated with anti-Smad4 (Abcam, Cambridge, UK), anti-S100A4 (BD Pharmingen, San Diego, CA) and followed by Alexa Fluor 488- and 594-conjugated secondary antibodies (Jackson Immuno Research Labs, Carlsbad, CA). After staining, bright field or fluorescence microscopy was performed under a microscope (DP71, OLYMPUS).

### RNA sequencing analysis

RNA-sequencing analyses were performed in DSS-induced acute colitis tissues from Smad4^fl/fl^ and S100A4^Smad4-/-^ mice. Total RNA was extracted with RNeasy Mini Kit (QIAGEN, Dusseldorf, Germany). Then, the RNA-sequencing analyses were performed on the BGISEQ-500 sequencer platform by BGI (Shenzhen, China). Using the principle of component analysis for the Stats package and plots with the ggplot2 package in R (version 3.5). After removing adaptor sequences, the raw transcriptomic reads were mapped to the C57BL/6 genome using HISAT40/Bowtie241 tools, including low-quality and polyN sequence reads. Using RESM software to perform Normalization, the threshold of padj<0.05, and the absolute value of log2 Ratio ≥ 1 to screen significantly differentially expressed genes (DEGs). The Kyoto Encyclopedia of Genes and Genomes (KEGG) enrichment analysis was performed using phyper in R. Making use of the Dr. Tom network platform of BGI to obtain the data mining.

### Quantitative real-time polymerase chain reaction (qPCR)

Using Trizol (TransGen Biotech, Beijing, China) to extract the total RNA, the 500 ng of RNA was reverse-transcribed to cDNA with a Quantscript RT kit (TIANGEN, Beijing, China). We also make use of the SuperReal PreMix Plus (SYBR Green) kit (TIANGEN, Beijing, China) to perform Quantitative reverse transcription according to the manufacturer's instructions. mRNA data are expressed relative to the reference gene. The gene-specific primers used are listed below in Table S1.

### Colony formation assay

For the colony formation assay, 1x10^3^ Sh-NC and Sh-Smad4 Raw264.7 cells were seeded onto each well of a 6-well plate and cultured for 2 weeks. Cells were fixed with 4% paraformaldehyde, stained with 0.5% crystal violet for 20 min, and then washed with cold PBS. The inverted plates were photographed and the number of colonies was counted.

### Transwell migration assays

1x10^5^ Raw264.7 cells were seeded into each chamber (Corning, 8 μm pore size, USA). Then, 100 μL of FBS-free DMEM and 500 μL of DMEM supplemented with 10% FBS were added to the upper and lower chambers, respectively. The migrating cells were then fixed, stained, and observed under a microscope after they were treated with IL-4 and IL-13 for 48 h.

### Cell scratch test

The Raw264.7 cells were stimulated with IL-4 and IL-13 for 48h, then, they were seeded onto 6-well plates at a density of 2×10^5^ cells per well. when cultured reaching 90% confluence, an artificial wound was created by scratching the surface using the tip of a P-200 pipette, and cells were cultured in a serum-free medium. The healing of cell scratches was observed and documented using a microscope (TS100, Nikon, Tokyo, Japan) at 0,24 and 48h.

### Flow cytometry

After IL-4 and IL-13 stimulated the Raw264.7 cells for 48h, the cell suspension was collected and stained with the mouse-specific mAbs: PerCP/Cy5.5-labeled anti-PD1 (clone M1/70). It was purchased from Biolegend (San Diego, CA) and used at a 0.2 mg/ml concentration. Cells were collected on a FACSCalibur (BD Biosciences, San Diego, CA) and analyzed by FlowJo software (TreeStar, Ashland, OR).

### Cell proliferation and viability assay

To obtain conditioned media, 1×10^6^ Sh-Smad4 Raw264.7 cells or Sh-NC Raw264.7 cells were seeded into 6 cm culture plates respectively, following treated with IL-4, IL-13, and BMS-30940. After 48 h, the supernatant was collected and centrifuged at 12,000×*g* for 10 min. 5×10^3^ MC38 cells were seeded into 96-well plates and cultured in a conditioned medium containing 50% supernatant and 50% complete media. The proliferative activity was detected by MTT according to the manufacturer's protocols. The OD value of cells was analyzed at 24 h.

### Western blotting

Cell extracts were analyzed using the following primary antibodies: anti-Smad4, anti-Fabp2, and anti-SCD1 (Santa Cruz Biotechnology, CA), anti-Arg1 (ImmunoWay, Newark, DE), and anti-β-actin (Cell Signaling, Danvers, MA). HRP-conjugated goat anti-rabbit IgG or goat anti-mouse were used as secondary antibodies.

### Lipid droplet staining with Nile red

The Raw264.7 cells were cultured in a medium supplemented with oleate (0.2 mM, Sigma, Darmstadt, Germany). Cells were washed with phosphate-buffered saline and stained with Nile red (Invitrogen, USA) at 37°C for 25 min. The quantification of Nile red content was measured by flow cytometry.

### Isolation of monocyte-derived macrophages

Bone marrow cells were isolated from the femur and tibia of 8-week-old male Lyz^Smad4-/-^ and Smad4^fl/fl^ mice. To obtain macrophage colony-stimulating factor (mCSF) from fibroblast conditioned medium (FCM), L929 fibroblasts were cultured in DMEM medium with 10% FBS for 3 days. The supernatant FCM was collected on the third day and stored at -80 ^°^C. Bone marrow cells were cultured in a DMEM medium containing 10% FBS and 20% FCM for 6 days to generate Bone marrow-derived macrophages (BMDMs). By the 7th day, the mature macrophages were all adherent cells.

### Mouse model of MC38 cell line transplanted tumors

Six-eight-week-old female Smad4^flox/flox^ and Lyz^Smad4-/-^ mice (n = 4) were selected. MC38 cells (7×10^5^) were inoculated subcutaneously into each mouse. On day 3, a group of the Lyz^Smad4-/-^ mice were injected intraperitoneally with Fabp inhibitor (BMS-309403) (10 mg/kg, once in two days), and the other mice were injected with the same volume of PBS at the same time. Tumor volumes were determined by caliper measurement using the formula V = (length × width^2^)/2. The mice were observed for 10-14 days the tumor of the mice was taken out, photographed, and weighed.

### Clinical samples

Human CRC tissue and adjacent non-tumor colon tissue samples were obtained from CRC patients at Peking University People's Hospital with informed consent. Transcriptome sequencing data from patients with CRC tumor tissues and normal tissues (n = 154) were collected at the Dazhou Central Hospital in our study. The Ethics Committee of Beijing Jiaotong University approved the use of human specimens in accordance with the Declaration of Helsinki.

### Public database analysis

Gene expression data (GSE75214, GSE32323) were downloaded from Gene Expression Omnibus (GEO) and analyzed using the online analysis tool GCBI website. Overall survival analysis was obtained from the Kaplan Meier-plotter dataset. Timer2.0 database was used for analyzing gene expression and correlations across cancers.

### Statistical analysis

All the data were analyzed by GraphPad Prism 8.2 software, and the results were represented by mean ± SEM. The difference between the two groups was compared by two-tailed unpaired Student's t-test. Multiple comparisons were made using two-way ANOVA analysis. The data are representative of at least three independent experiments. P < 0.05 was considered statistically significant.

## Supplementary Material

Supplementary figures and tables.

## Abbreviations

AOM: Azoxymethan
BMDMs: Bone marrow-derived macrophages
CAC: Colitis-associated cancer
CRC: Colorectal cance
CD: Crohn's diseas
DSS: Dextran sodium sulfate
DMEM: Dulbecco's modified Eagle's mediu
FAO: Fatty acid oxidation
Fabp2: Fatty acid binding protein 2
FCM: Fibroblast conditioned mediu
GO: Gene Ontology
H&E: Hematoxylin and eosin
IBD: Inflammatory bowel diseases
KEGG: Kyoto Encyclopedia of Genes and Genomes
PD-1: Promoted programmed cell death protein 1
S100A4: S100 calcium binding protein A4
Smad4: Sma mothers against decapentaplegic homologue 4
SCD1: Stearoyl-CoA desaturase-1
TME: Tumor microenvironment
TAMs: Tumor-associated macrophage
UC: Ulcerative colitis

## References

[1] Coussens LM, Werb Z (2002). Inflammation and cancer. Nature.

[2] Manjili SH, Isbell M, Ghochaghi N, Perkinson T, Manjili MH (2022). Multifaceted functions of chronic inflammation in regulating tumor dormancy and relapse. Seminars in cancer biology.

[3] Qiu H, Cao S, Xu R (2021). Cancer incidence, mortality, and burden in China: a time-trend analysis and comparison with the United States and United Kingdom based on the global epidemiological data released in 2020. Cancer communications (London, England).

[4] Cai J, Sun L, Gonzalez FJ (2022). Gut microbiota-derived bile acids in intestinal immunity, inflammation, and tumorigenesis. Cell host & microbe.

[5] Rogler G (2014). Chronic ulcerative colitis and colorectal cancer. Cancer letters.

[6] Bopanna S, Ananthakrishnan AN, Kedia S, Yajnik V, Ahuja V (2017). Risk of colorectal cancer in Asian patients with ulcerative colitis: a systematic review and meta-analysis. The lancet Gastroenterology & hepatology.

[7] Chen S, Zhou Z, Li Y, Du Y, Chen G (2023). Application of single-cell sequencing to the research of tumor microenvironment. Frontiers in immunology.

[8] Huang J, Wu Q, Geller DA, Yan Y (2023). Macrophage metabolism, phenotype, function, and therapy in hepatocellular carcinoma (HCC). Journal of translational medicine.

[9] Wang T, Du G, Wang D (2021). The S100 protein family in lung cancer. Clinica chimica acta; international journal of clinical chemistry.

[10] Zhang W, Ohno S, Steer B, Klee S, Staab-Weijnitz CA, Wagner D (2018). S100a4 Is Secreted by Alternatively Activated Alveolar Macrophages and Promotes Activation of Lung Fibroblasts in Pulmonary Fibrosis. Frontiers in immunology.

[11] Fei F, Qu J, Zhang M, Li Y, Zhang S (2017). S100A4 in cancer progression and metastasis: A systematic review. Oncotarget.

[12] Liu L, Qi L, Knifley T, Piecoro DW, Rychahou P, Liu J (2019). S100A4 alters metabolism and promotes invasion of lung cancer cells by up-regulating mitochondrial complex I protein NDUFS2. The Journal of biological chemistry.

[13] Takenaga K, Nakanishi H, Wada K, Suzuki M, Matsuzaki O, Matsuura A, Endo H (1997). Increased expression of S100A4, a metastasis-associated gene, in human colorectal adenocarcinomas. Clinical cancer research: an official journal of the American Association for Cancer Research.

[14] Kim JH, Kim CN, Kim SY, Lee JS, Cho D, Kim JW, Yoon SY (2009). Enhanced S100A4 protein expression is clinicopathologically significant to metastatic potential and p53 dysfunction in colorectal cancer. Oncology reports.

[15] Boye K, Nesland JM, Sandstad B, Mælandsmo GM, Flatmark K (2010). Nuclear S100A4 is a novel prognostic marker in colorectal cancer. European journal of cancer (Oxford, England: 1990).

[16] Huang LY, Xu Y, Cai GX, Guan ZQ, Sheng WQ, Lu HF (2011). S100A4 over-expression underlies lymph node metastasis and poor prognosis in colorectal cancer. World journal of gastroenterology.

[17] Zhang J, Hou S, Gu J, Tian T, Yuan Q, Jia J (2018). S100A4 promotes colon inflammation and colitis-associated colon tumorigenesis. Oncoimmunology.

[18] Zhang J, Jiao Y, Hou S, Tian T, Yuan Q, Hao H (2017). S100A4 contributes to colitis development by increasing the adherence of Citrobacter rodentium in intestinal epithelial cells. Scientific reports.

[19] McCarthy AJ, Chetty R (2018). Smad4/DPC4. Journal of clinical pathology.

[20] Liao X, Ruan X, Yao P, Yang D, Wu X, Zhou X (2023). LncRNA-Gm9866 promotes liver fibrosis by activating TGFβ/Smad signaling via targeting Fam98b. Journal of translational medicine.

[21] Chandiran K, Cauley LS (2023). The diverse effects of transforming growth factor-β and SMAD signaling pathways during the CTL response. Frontiers in immunology.

[22] Loevenich LP, Tschurtschenthaler M, Rokavec M, Silva MG, Jesinghaus M, Kirchner T (2022). SMAD4 Loss Induces c-MYC-Mediated NLE1 Upregulation to Support Protein Biosynthesis, Colorectal Cancer Growth, and Metastasis. Cancer research.

[23] Hanna DN, Smith PM, Novitskiy SV, Washington MK, Zi J, Weaver CJ (2022). SMAD4 Suppresses Colitis-associated Carcinoma Through Inhibition of CCL20/CCR6-mediated Inflammation. Gastroenterology.

[24] Chen L, Li J, Zhang J, Dai C, Liu X, Wang J (2015). S100A4 promotes liver fibrosis via activation of hepatic stellate cells. J Hepatol.

[25] Liu S, Zhang H, Li Y, Zhang Y, Bian Y, Zeng Y (2021). S100A4 enhances protumor macrophage polarization by control of PPAR-γ-dependent induction of fatty acid oxidation. Journal for immunotherapy of cancer.

[26] Wu Y, Liang M, Huang F, Cheng OH, Xiao X, Lee TH (2023). Notch Blockade Specifically in Bone Marrow-Derived FSP-1-Positive Cells Ameliorates Renal Fibrosis. Cells.

[27] Popov J, Caputi V, Nandeesha N, Rodriguez DA (2021). Microbiota-Immune Interactions in Ulcerative Colitis and Colitis Associated Cancer and Emerging Microbiota-Based Therapies. Int J Mol Sci.

[28] Zhang M, Li X, Zhang Q, Yang J, Liu G (2023). Roles of macrophages on ulcerative colitis and colitis-associated colorectal cancer. Frontiers in immunology.

[29] Mehla K, Singh PK (2019). Metabolic Regulation of Macrophage Polarization in Cancer. Trends Cancer.

[30] Su P, Wang Q, Bi E, Ma X, Liu L, Yang M (2020). Enhanced Lipid Accumulation and Metabolism Are Required for the Differentiation and Activation of Tumor-Associated Macrophages. Cancer research.

[31] Cao M, Zhang Y, Chen D, Zhong J, Zhang X, Yang L (2023). Polymorphism in genes encoding two fatty acid binding proteins increases risk of ischemic stroke in a Chinese Han population. Frontiers in genetics.

[32] Yan J, Horng T (2020). Lipid Metabolism in Regulation of Macrophage Functions. Trends in cell biology.

[33] Tao Y, Xu L, Liu X, Wang P, Wei S, Huang Y (2023). Chitosan-coated artesunate protects against ulcerative colitis via STAT6-mediated macrophage M2 polarization and intestinal barrier protection. International journal of biological macromolecules.

[34] Ogawa R, Yamamoto T, Hirai H, Hanada K, Kiyasu Y, Nishikawa G (2019). Loss of SMAD4 Promotes Colorectal Cancer Progression by Recruiting Tumor-Associated Neutrophils via the CXCL1/8-CXCR2 Axis. Clinical cancer research: an official journal of the American Association for Cancer Research.

[35] Kitamura T, Kometani K, Hashida H, Matsunaga A, Miyoshi H, Hosogi H (2007). SMAD4-deficient intestinal tumors recruit CCR1+ myeloid cells that promote invasion. Nature genetics.

[36] Itatani Y, Kawada K, Fujishita T, Kakizaki F, Hirai H, Matsumoto T (2013). Loss of SMAD4 from colorectal cancer cells promotes CCL15 expression to recruit CCR1+ myeloid cells and facilitate liver metastasis. Gastroenterology.

[37] Means AL, Freeman TJ, Zhu J, Woodbury LG, Marincola-Smith P, Wu C (2018). Epithelial Smad4 Deletion Up-Regulates Inflammation and Promotes Inflammation-Associated Cancer. Cellular and molecular gastroenterology and hepatology.

[38] Choi SH, Huang AY, Letterio JJ, Kim BG (2022). Smad4-deficient T cells promote colitis-associated colon cancer via an IFN-γ-dependent suppression of 15-hydroxyprostaglandin dehydrogenase. Frontiers in immunology.

[39] Wen Y, Zhu Y, Zhang C, Yang X, Gao Y, Li M (2022). Chronic inflammation, cancer development and immunotherapy. Frontiers in pharmacology.

[40] Wang X, Su S, Zhu Y, Cheng X, Cheng C, Chen L (2023). Metabolic Reprogramming via ACOD1 depletion enhances function of human induced pluripotent stem cell-derived CAR-macrophages in solid tumors. Nature communications.

[41] Wang S, Liu R, Yu Q, Dong L, Bi Y, Liu G (2019). Metabolic reprogramming of macrophages during infections and cancer. Cancer letters.

[42] Hossain F, Al-Khami AA, Wyczechowska D, Hernandez C, Zheng L, Reiss K (2015). Inhibition of Fatty Acid Oxidation Modulates Immunosuppressive Functions of Myeloid-Derived Suppressor Cells and Enhances Cancer Therapies. Cancer Immunol Res.

[43] Ockner RK, Manning JA (1974). Fatty acid-binding protein in small intestine. Identification, isolation, and evidence for its role in cellular fatty acid transport. The Journal of clinical investigation.

[44] Peng X, Zhang L, Wang Q, Cui X (2009). [Study on the relationship between FABP2 Ala54Thr polymorphism and the risk of non-alcoholic fatty liver diseases]. Wei sheng yan jiu = Journal of hygiene research.

[45] Huang X, Zhou Y, Sun Y, Wang Q (2022). Intestinal fatty acid binding protein: A rising therapeutic target in lipid metabolism. Progress in lipid research.

[46] Shi JH, Liu LN, Song DD, Liu WW, Ling C, Wu FX (2023). TRAF3/STAT6 axis regulates macrophage polarization and tumor progression. Cell death and differentiation.

[47] Delgado-Ramirez Y, Colly V, Gonzalez GV, Leon-Cabrera S (2020). Signal transducer and activator of transcription 6 as a target in colon cancer therapy. Oncology letters.

[48] Li YJ, Zhang C, Martincuks A, Herrmann A, Yu H (2023). STAT proteins in cancer: orchestration of metabolism. Nature reviews Cancer.

