# Supplementary material for: Smad4 Deficiency in S100A4+ Macrophages Enhances Colitis-associated Tumorigenesis by Promoting Macrophage Lipid Metabolism Augmented M2 Polarization
Source: Int J Biol Sci. 2024 Nov 11;20(15):6114–29. doi: 10.7150/ijbs.98529 (PMC11628331; doi:10.7150/ijbs.98529)
Supplement: Supplementary file 1 — Supplementary figures and tables. [file ijbsv20p6114s1.pdf]

Supplementary figures and tables

Figure S1

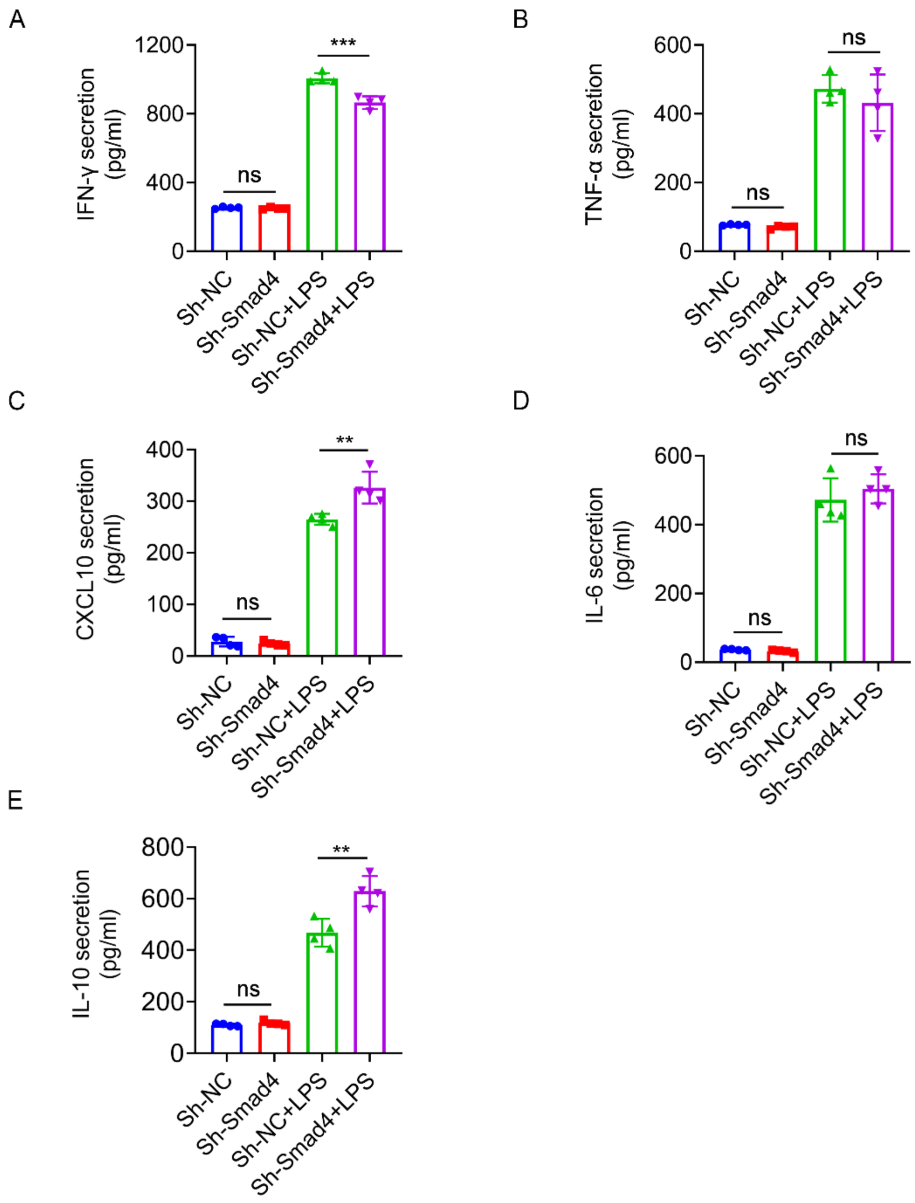

Figure S1. Effect of Smad4 deficiency on cytokine secretion of Raw264.7 cells. (A-E) Elisa analysis of (A) IFN-γ, (B) TNF-α, (C) CXCL10, (D) IL-6, and (E) IL-10 secretion in Raw264.7 cells following LPS (100 ng/mL) stimulation. $^{**}P < 0.01$, $^{***}P < 0.001$.

Figure S2

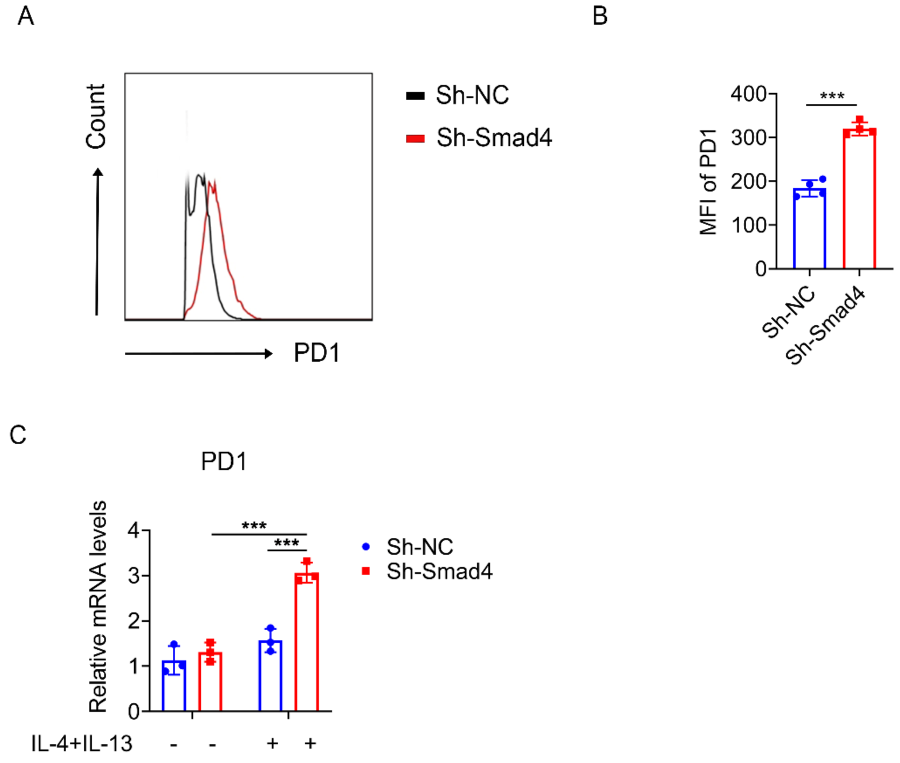

Figure S2. Smad4 deficiency in Raw264.7 promotes the expression of PD1. (A-B) The expression of PD1 in Sh-NC and Sh-Smad4 Raw264.7 cells was detected by flow cytometry. ***P < 0.001. (C) qPCR analysis of PD1 expression in Raw264.7 cells. ***P < 0.001.

Figure S3

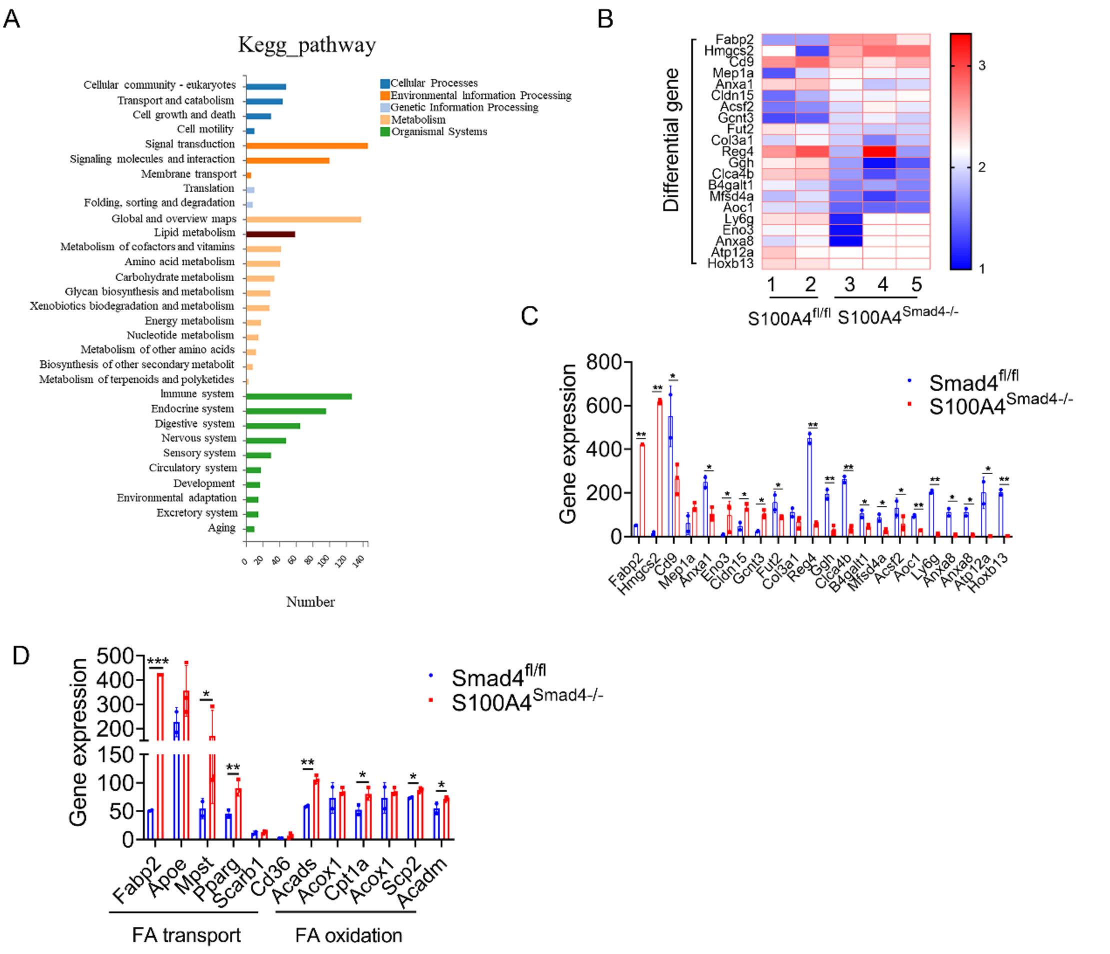

Figure S3. Smad4 deficiency in S100A4^{+} cells facilitates fatty acid metabolism. RNA sequencing analysis of differentially expressed genes (DEGs) between DSS-induced colitis tissues from Smad4$^{fl/fl}$ and S100A4$^{Smad4-/-}$ mice. (A) Kyoto Encyclopedia of Genes and Genomes (KEGG) analysis of DEGs. (B) Heatmap view of the most significant DEGs, and (C) analysis of fold change of DEGs. *$P < 0.05$, **$P < 0.01$. (D) Fold change of the most significant DEGs related to fatty acid metabolism. *$P < 0.05$, **$P < 0.01$.

Figure S4

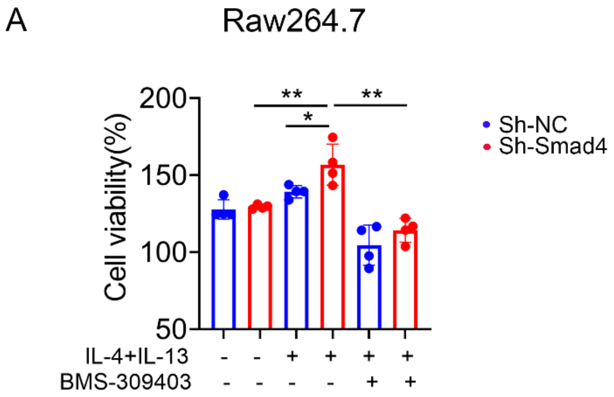

Figure S4. Smad4 deficiency in macrophage promoted proliferative activity of MC38 cells. (A) MC38 cells were treated with the supernatants from Raw264.7 cells following IL-4 (20 ng/ml), IL-13 (20 ng/ml), and BMS-309403 (40 μM) treatment, and proliferative activity of MC38 cells was detected by MTT. *P < 0.05, **P < 0.01.

Table S1. Real-time PCR primer sequences.

Gene	Species	Primer sequence (5'-3')
Actin	Mouse	F: CACCAGTTCGCCATGGATGACGA<br>R: ATACCTCTCTTGCTCTGGGCCTCG
Arg1	Mouse	F: CTCCAAGCCAAAGTCCTTAGAG<br>R: AGGAGCTGTCATTAGGGACATC
IL-10	Mouse	F: GCTCTTACTGACTGGCATGAG<br>R: CGCAGCTCTAGGAGCATGTG
YM1	Mouse	F: CTCAACCTGGACTGGCAGTA<br>R: CTGCTCCTGTGGAAGTGAGT
Fabp2	Mouse	F: GTCTAGCAGACGGAACGGAG<br>R: GTCTAGCAGACGGAACGGAG
Scara1	Mouse	F: GTG CTG TCT TCT TTA CCA GCA A<br>R: GCT GTC ATT GAA CGT GCG TC
FASN	Mouse	F: GGA GGT GGT GAT AGC CGG TAT<br>R: TGG GTA ATC CAT AGA GCC CAG
Scarb1	Mouse	F: TGA TGG AGA GCA AGC CTG TG<br>R:TGA TGG AGA GCA AGC CTG TG
SCD1	Mouse	F: TTCTTGCGATACACTCTGGTGC<br>R:CGGGATTGAATGTTCTTGTCGT
iNOS	Mouse	F:CGGAGATCAATGTGGCTGTG<br>R:GAAGGACTCTGAGGCTGTGT

Table S2. Details of primary materials and antibodies used in the study.

	Catalogue number	Identifier
Materials		
DMEM	06-1055-57-1ACS-1	BI
RMPI-1640	SH30809.01	Hyclone
FBS	A31608-02	Gibco
Pen-Strep Solution	03-031-1B	BI
AOM	A5486	Sigma-Aldrich
DSS	160110	MP Biomedicals
FastKing RT Kit (With gDNase)	KR116	TIANGEN
SYBR Green qPCR Master Mix	HY-K0501-5	MCE
Fixation & Permeabilization Buffer	88-8824-00	Invitrogen
IFN-γ ELISA kit	SEA049Mu	Cloud-Clone
TNF-α ELISA kit	SEA133Mu	Cloud-Clone
IL-2 ELISA kit	SEA073Mu	Cloud-Clone
Antibodies		
anti-Mouse CD11b	550282	BD Biosciences
anti-Mouse F4/80	123101	BioLegend
anti-Mouse Gr1	550291	BD Biosciences
Mouse monoclonal anti-Fabp2	sc-374482	Santa Cruz
Mouse monoclonal anti-Ki67	550609	BD Biosciences
Phospho-STAT6 (Tyr641) Antibody	AF3301	Affinity
STAT6 Antibody	AF6302	Affinity
Mouse monoclonal anti-β-actin	sc-47778	Santa Cruz
Experimental models: cell lines		
Raw264.7	TIB-71	American Type Culture Collection
L929	CCL-1	American Type Culture Collection

MC38	m032	icell
Experimental models: organisms/strains		
C57BL/6		National Institute of Biological Sciences
Mouse: Smad4$^{fl/fl}$	1	The Jackson Laboratory
Mouse: Lyz-Cre	2	The Jackson Laboratory
Mouse: S100A4-Cre	3	The Jackson Laboratory
Software and algorithms		
ImageJ	https://imagej.nih.gov/ij	NIH
Prism 6	http://www.graphpad.com/scientific-software/prism	GraphPad
FlowJo	https://www.flowjo.com	FlowJo
GEO Database	http://www.ncbi.nlm.nih.gov/go	Open source
Timer2.0	http://timer.cistrome.org/	Open source

Reference

1. Garcia-Carracedo D, Yu CC, Akhavan N, et al. Smad4 loss synergizes with TGFα overexpression in promoting pancreatic metaplasia, PanIN development, and fibrosis. *PLoS One* 2015;10(3):e0120851. doi: 10.1371/journal.pone.0120851 [published Online First: 20150324]
2. Clausen BE, Burkhardt C, Reith W, et al. Conditional gene targeting in macrophages and granulocytes using LysMcre mice. *Transgenic Res* 1999;8(4):265-77. doi: 10.1023/a:1008942828960
3. Tsutsumi R, Xie C, Wei X, et al. PGE2 signaling through the EP4 receptor on fibroblasts upregulates RANKL and stimulates osteolysis. *J Bone Miner Res* 2009;24(10):1753-62. doi: 10.1359/jbmr.090412
